# Deciphering lung adenocarcinoma prognosis and immunotherapy response through an AI‐driven stemness‐related gene signature

**DOI:** 10.1111/jcmm.18564

**Published:** 2024-07-24

**Authors:** Bicheng Ye, Ge Hongting, Wen Zhuang, Cheng Chen, Shulin Yi, Xinyan Tang, Aimin Jiang, Yating Zhong

**Affiliations:** ^1^ School of Clinical Medicine Yangzhou Polytechnic College Yangzhou China; ^2^ Department of Respiratory and Critical Care Medicine Huai'an Hospital Affiliated to Yangzhou University (The Fifth People's Hospital of Huai'an) Huai'an China; ^3^ Huai'an Second People's Hospital Affiliated to Xuzhou Medical University Huai'an Jiangsu China; ^4^ Department of Nursing Jiangsu Vocational College of Medicine Yancheng China; ^5^ Department of Urology, Changhai Hospital Naval Medical University (Second Military Medical University) Shanghai China; ^6^ Department of Oncology Shuyang County Hospital of Traditional Chinese Medicine Suqian China

**Keywords:** artificial intelligence, immunotherapy, machine learning, prognosis, single‐cell RNA sequencing, stemness

## Abstract

Lung adenocarcinoma (LUAD) is a leading cause of cancer‐related deaths, and improving prognostic accuracy is vital for personalised treatment approaches, especially in the context of immunotherapy. In this study, we constructed an artificial intelligence (AI)‐driven stemness‐related gene signature (SRS) that deciphered LUAD prognosis and immunotherapy response. CytoTRACE analysis of single‐cell RNA sequencing data identified genes associated with stemness in LUAD epithelial cells. An AI network integrating traditional regression, machine learning, and deep learning algorithms constructed the SRS based on genes associated with stemness. Subsequently, we conducted a comprehensive exploration of the connection between SRS and both intrinsic and extrinsic immune environments using multi‐omics data. Experimental validation through siRNA knockdown in LUAD cell lines, followed by assessments of proliferation, migration, and invasion, confirmed the functional role of CKS1B, a top SRS gene. The SRS demonstrated high precision in predicting LUAD prognosis and likelihood of benefiting from immunotherapy. High‐risk groups classified by the SRS exhibited decreased immunogenicity and reduced immune cell infiltration, indicating challenges for immunotherapy. Conversely, in vitro experiments revealed CKS1B knockdown significantly impaired aggressive cancer phenotypes like proliferation, migration, and invasion of LUAD cells, highlighting its pivotal role. These results underscore a close association between stemness and tumour immunity, offering predictive insights into the immune landscape and immunotherapy responses in LUAD. The newly established SRS holds promise as a valuable tool for selecting LUAD populations likely to benefit from future clinical stratification efforts.

## 1 INTRODUCTION

The global incidence of lung adenocarcinoma (LUAD) is on the rise.^1^, ^2^ Despite the ongoing use of traditional treatments like surgery and chemotherapy, burgeoning molecular knowledge from recent technological breakthroughs has enabled the discovery of molecular targets for LUAD, paving the way for targeted therapy development. A significant segment of LUAD patients carrying genomic alterations such as EGFR, ALK, and HER2, show considerable improvement with these targeted approaches.^3^ Despite this progress, LUAD continues to pose a formidable global health challenge, with an overall survival (OS) rate not exceeding five years.^4^ Furthermore, the resistance exhibited by LUAD patients to extant therapies exacerbates the imperative to seek and formulate new efficacious interventions. The introduction of immunotherapy has heralded a revolutionary epoch in oncological management, demonstrating extraordinary and auspicious therapeutic outcomes across a diverse spectrum of solid neoplasms. Therapies aimed at PD‐1/PD‐L1 and CTLA‐4 have substantiated their therapeutic merit and garnered approval for clinical utilisation.^5^ Nonetheless, the proportion of LUAD patients who accrue benefits from such treatments remains a minority.^6^, ^7^ This reality has catalysed a wave of research dedicated to unlocking the potential of the immune system and eliciting a cogent response to immunotherapy.

The eventual death of cancer patients often aligns with the progression and metastasis of the tumour, a process for which cancer stem cells (CSCs) are considered the key driving factor.^8^ Although CSCs only comprise an insignificant proportion in the tumour mass, they share similar characteristics with normal stem cells, enabling them to initiate and promote tumour growth.^9^, ^10^ Intrinsic or acquired resistance to radiotherapy and chemotherapy is prominently found in CSCs, serving as a major cause for many recurring, spreading, or even fatal tumours.^11^, ^12^ Additionally, CSCs have the ability to evade immune control. They can change the tumour microenvironment (TME) into an immune‐suppressive and tumour‐promoting environment.^13^

Single‐cell RNA sequencing (scRNA‐seq) offers an intricate examination of the heterogeneity within the TME at high resolution.^14^ By harnessing the high‐resolution capabilities of this method, researchers can precisely characterise cell populations within tumour tissues–populations that could remain concealed due to the dominance of certain cell types when employing traditional bulk sequencing techniques.^15^ Utilising the sophisticated computational framework (CytoTRACE), developed by Gulati et al., we are equipped to precisely characterise cancer stemness and pinpoint genes correlated with stemness at the single‐cell resolution.^16^ This enhances our investigation into the effects of stemness on immune checkpoint inhibitors (ICIs). Artificial intelligence (AI) plays a pivotal role in the construction of robust predictive models grounded in large‐scale data sets. Leveraging these cutting‐edge technologies enables a more profound exploration of the multifaceted mechanisms underlying therapy resistance at genes correlated with stemness, thereby unveiling further insights to enhance the efficacy of immunotherapy.

In our research, we utilised scRNA‐seq to identify genes associated with stemness in LUAD epithelial cells and construct SRS based on a novel AI network. This signature was evaluated through the intrinsic and extrinsic immune landscapes from a multi‐omics standpoint and corroborated by in vitro experiments, thus providing a novel vantage point on immunotherapy and prognosis in LUAD.

## 2 METHODS

### 2.1 Analysis of the scRNA‐seq data

A dataset composed of scRNA‐seq for 58 samples of LUAD was accumulated, strictly adhering to the quality control protocol as delineated in the research.^17^ For subsequent investigation, 208,506 cells, chosen after rigorous selection, were subjected to the Seurat R package.^18^ Gene expression levels underwent normalisation using the LogNormalize method, leveraging a scale factor of 10,000. The top 2000 genes exhibiting maximum variable expression were identified and subjected to scaling before principal component analysis (PCA). Batch effects were mitigated using the Harmony R package.^19^ Employing the Harmony and Seurat packages, all procedures, including NormalizeData, FindVariableFeatures, ScaleData, RunPCA, FindNeighbors, FindClusters, and RunUMAP, were executed with accuracy. Furthermore, singular cell annotations were procured, stemming from the originating study premised on the acquired single‐cell data.^17^

### 2.2 CytoTRACE Analysis

CytoTRACE stands as a robust computational framework for inferring cellular differentiation states from scRNA‐seq data, with validation against extensive datasets, and outstrips earlier computational methods for stemness estimation.^20^ In analysing the stemness of malignant cells, the CytoTRACE R package was utilised to calculate CytoTRACE scores, which range from 0 to 1. A score closer to 1 is indicative of greater stemness or a lesser degree of differentiation, while a lower score suggests the opposite. A gene is considered stemness‐related when the absolute value of its correlation coefficient is greater than 0.5.

### 2.3 Analysis of the Bulk RNA‐seq data

The acquisition of transcriptome data and clinical files pertaining to LUAD was achieved from the Cancer Genome Atlas (TCGA) database (https://portal.gdc.cancer.gov), which consists of RNA sequencing data, mutation information, and survival data. In the interest of model validation, six additional datasets were sourced from the Gene Expression Omnibus (GEO) database (http://www.ncbi.nlm.nih.gov/geo).

The datasets GSE126045 (non‐small cell lung cancer, NSCLC),^21^ OAK (NSCLC),^22^ POPLAR (NSCLC),^23^ NG (NSCLC),^24^ PRJNA482620 (glioblastoma multiforme, GBM),^25^ GSE91061 (melanoma),^26^ Braun (renal cell carcinoma, RCC),^27^ and phs000452 (melanoma)^28^ were downloaded. Within each dataset, the SRS was computed to predict the response to immunotherapy.

To ensure a standardised data format from the outset of the analysis, all data sets underwent log transformation. To correct for potential batch effects, we applied the combat function from the sva R package.

### 2.4 Development of signatures using an artificial intelligence network

We aimed to develop an accurate and stable SRS for predicting the prognosis of LUAD patients. To this end, we constructed an artificial intelligence network based on 429 algorithm combinations, integrating 27 algorithms from traditional regression, machine learning and deep learning. These algorithms included stepwise Cox, random survival forest (RSF), gradient boosting machine (GBM), supervised principal components (SuperPC), oblique random survival forests (obliqueRSF), conditional random forests (CForest), gradient boosting with component‐wise linear models (GLMBoost), gradient boosting with regression trees (BlackBoost), recursive partitioning and regression trees (Rpart), parametric survival model (Survreg), Ranger, conditional inference trees (Ctree), least absolute shrinkage and selection operator (LASSO), partial least squares regression for Cox (plsRcox), survival support vector machine (survival‐SVM), Ridge, elastic network (Enet), deephit survival neural network (DeepHit), deepsurv survival neural network (DeepSurv), cox‐time survival neural network (CoxTime), extreme gradient boosting (XGBoost), Boruta, logistic‐hazard survival neural network (Logistic‐Hazard), PC‐hazard survival neural network (PC‐hazard), akritas conditional non‐parametric survival estimator (Akritas), Coxboost, and variable selection oriented LASSO bagging algorithm (VSOLassoBag). Within the TCGA cohort, we engaged 429 distinct algorithm combinations to craft predictive models utilising the stemness‐related genes. The predictive efficacy of each algorithmic combination was appraised by the concordance index (C‐index) across all validation cohorts. The selection of the optimal algorithm combination was predicated on the attainment of the maximal mean C‐index. Patients were stratified into high‐ and low‐risk categories based on the optimal cut‐off value ascertained through the survminer R package. The source code and specific parameters of this artificial intelligence network can be found at the following GitHub repository: https://github.com/yebicheng/artificial‐intelligence‐network.

### 2.5 Functional annotation of the SRS score

We performed gene set variation analysis (GSVA) and gene set enrichment analysis (GSEA) using the MSigDB database with the GSVA^29^ and clusterprofiler^30^ R packages. We also used Metascape for enrichment analysis.^31^

### 2.6 Evaluation of immune infiltration utilising CIBERSORT

CIBERSORT, a deconvolution algorithm based on gene expression profiles, utilises support vector regression to infer the proportions of cell types within bulk cancer samples that comprise mixed cellular populations.^32^ Employing this method, CIBERSORT estimated the proportions of 22 infiltrating immune cell types using normalised gene expression data. The immune infiltration proportions determined through CIBERSORT originated from the pan‐cancer immune landscape project conducted by Thorsson et al.^33^

### 2.7 Tumour‐infiltrating lymphocyte fraction, leukocyte fraction, and lymphocyte fraction analyses

Within the cohort from the TCGA, evaluations of tumour‐infiltrating lymphocytes (TILs) levels based on genomic data were juxtaposed with those derived from haematoxylin and eosin (H&E)‐stained image assessments. This comparative analysis drew on data from Thorsson et al. and Saltz et al.^33^, ^34^ The method utilised by Saltz et al. for analysing TILs involves a deep learning‐based lymphocyte classification system that employs convolutional neural networks (CNNs). The genomic appraisal of TILs fractions necessitated the product of the combined lymphocyte proportion in the immune compartment, as ascertained by the CIBERSORT method, and the leukocyte fraction deduced from DNA methylation analysis.

### 2.8 Immune signature evaluation

The immune infiltration scores were derived from a pan‐cancer study of the TCGA conducted by Danaher et al.^35^ Computation of these scores was based on the expression of 60 specific marker genes capable of identifying 14 different immune cell populations. The robustness of these findings was corroborated, evidencing high reproducibility and agreement with results from immunohistochemistry and flow cytometry. Additionally, twenty‐nine classic immune signatures were sourced from He et al. The abundance of these immune signatures in each specimen was measured with the GSVA^29^ package in R. The cytolytic activity score (CYT) was defined as the geometric mean of the expression levels of granzyme A (GZMA) and perforin 1 (PRF1).^36^

### 2.9 Calculation of the immunogenomic indicator

Immunogenomic indicators were sourced from the pan‐cancer immune landscape undertaking by Torsson et al.^33^ The metrics ‘n_segs’ and ‘frac_altered’ represent the total count of segments in each sample's copy number profile and the fraction of bases that vary from the established ploidy level, respectively. Aneuploidy scores were formulated by summing the number of amplified or deleted genomic arms. Diversity scores for T‐cell receptors (TCR), reflected through Shannon entropy and richness, as well as B‐cell receptor (BCR) diversity scores, similarly measured by Shannon entropy and richness, were derived from RNA‐seq datasets of cancer. The tumour mutational burden (TMB) of each tumour was calculated using the maftools package in R.

### 2.10 Decoding the mutational signatures in the genome

The magtools R package was employed to perform nonnegative matrix factorization (NMF) analysis on mutations classified according to 96 trinucleotide contexts within pan‐cancer samples from the TCGA database. The resultant mutational signatures were assessed against the Catalogue of Somatic Mutations in Cancer (COSMIC) database using measures of cosine similarity.

### 2.11 Oncogenic Pathway Scores

Sanchez‐Vega et al.^37^ dentified ten canonical oncogenic pathways, encompassing 187 oncogenes. Enrichment scores for individual pathways in each sample were determined utilising the GSVA package in R.

### 2.12 Cell lines culture

The A549 and H1299 lung adenocarcinoma (LUAD) cell lines were procured from the Institute of Biochemistry and Cell Biology of the Chinese Academy of Sciences in Shanghai, China. The A549 cells were maintained in Dulbecco's Modified Eagle Medium (DMEM), while the H1299 cells were cultured in Roswell Park Memorial Institute (RPMI) 1640 medium. Both mediums were augmented with 10% foetal bovine serum (FBS) and 1% antibiotics (100 units/mL penicillin and 100 μg/mL streptomycin).

### 2.13 Transfection of small interfering RNA

Small interfering RNA (siRNA) transfection was carried out with the Lipo2000 reagent (Invitrogen, Shanghai, China), strictly following the prescribed protocols of the manufacturer. Generally, coverslips within six‐well plates were utilised for the deposition of A549 and H1299 cells, and the transfection of plasmid or siRNA was performed on the subsequent day. CKS1B shRNA target sequences were: shRNA1: 5′‐GGTCCATTATATGATCCAT‐3′; shRNA2: 5′‐GATGGGTCCATTATATGAT‐3′.

### 2.14 Reverse transcription‐quantitative polymerase chain reaction

We extracted RNA from both cells and tissues using Trizol reagent (Invitrogen) and performed reverse transcription using SuperScript II reverse transcriptase (Invitrogen) according to the manufacturer's protocol. The primer sequences used for CKS1B and β‐actin were as follows: CKS1B Forward: 5’‐TATTCGGACAAATACGACGACG‐3′; CKS1B Reverse: 5’‐CGCCAAGATTCCTCCATTCAGA‐3′; β‐actin Forward: 5’‐AGTGTGAGCGTGGACATCCGCAA‐3′; β‐actin Reverse: 5′‐GGA ATCCACATCTGCTGGAAGGTGGAC‐3′.

### 2.15 Colony formation

A quantity of five thousand cells was introduced into each well of a 6‐well plate as part of the colony formation experiment, and conventional growth medium was introduced, later substituted after one week. Methanol was utilised for a period of 15 minutes after the colonies had reached maturity within a two‐week span, followed by staining with 0.1% crystal violet (Sigma) for 30 minutes. Following this procedure, the resultant clones were quantified to determine the colony‐forming capability of the clones.

### 2.16 Ethynyl deoxyuridine

The labelling and staining of cells with 5‐ethynyl‐2′‐deoxyuridine (EdU) were conducted using an EdU Cell Proliferation Detection Kit from RiboBio, Guangzhou, China. Cells were seeded into 96‐well plates at 5 × 10^3 cells/well and treated with 50 μM EdU labeling medium 48 hours after transfection. Following a 2‐hour incubation at 37°C and 5% CO_2_, the cells were fixed with 4% paraformaldehyde and permeabilized with 0.5% Triton X‐100 before staining with the anti‐EdU working solution. Nuclear staining was achieved using diamidino‐2‐phenylindole (DAPI). The proportion of EdU‐positive cells was quantified using fluorescence microscopy.

### 2.17 Wound‐Healing assay

Cells were seeded in 6‐well plates and cultured until they reached 90–100% confluence. A fine pipette tip was then used to create a scratch in the confluent cell monolayer, after which the cells were gently washed twice with phosphate‐buffered saline (PBS). Microscopic images were taken at identical positions in each well at 0 and 48 hours using an Olympus microscope (Tokyo, Japan). Wound closure was quantified as the percentage of initial wound confluence using ImageJ software.

### 2.18 Invasion and migration assays

Invasion and migration assays were performed using the Transwell system (Corning, New York, NY, USA) with 24‐well plates and 8 μm pores. For migration assays, 5 × 10^4 post‐transfection cells were seeded into the upper chambers containing 350 μL of serum‐free medium, while 700 μL of medium supplemented with 10% foetal bovine serum (FBS) was added to the lower chambers. Invasion assays involved the use of Transwell membranes pre‐coated with Matrigel (Sigma‐Aldrich). After a 16‐hour incubation, non‐invading cells on the upper membrane surface were removed, and cells that migrated to the lower surface were fixed with methanol and stained with 0.1% crystal violet. Images were captured using an inverted Olympus microscope (Tokyo, Japan).

### 2.19 Statistical Analysis

We evaluated the correlation between the SRS and OS using the Kaplan–Meier approach, using the log‐rank test to compare survival curves. C‐indexes were computed to measure the prognostic precision of the SRS compared to other parameters. The Wilcoxon test was employed for statistical comparison between two distinct groups. All the statistical computations were carried out with R software, with two‐sided *p* values. A *p* value less than 0.05 was considered as statistically significant.

## 3 RESULTS

### 3.1 Identification of stemness‐related genes

**FIGURE 1:**
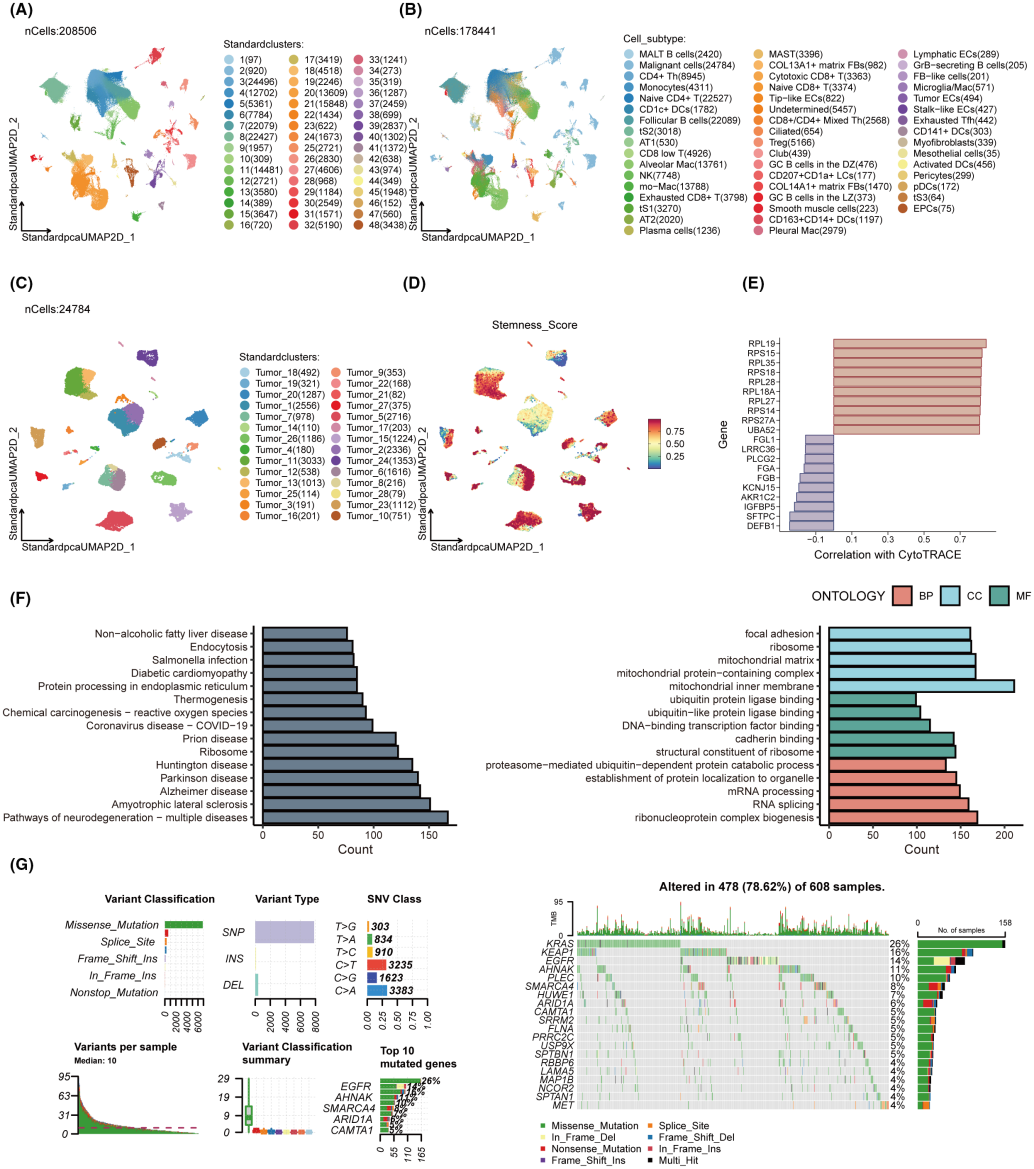
Identification of stemness‐related genes. (A, B) Cluster annotation and cell type identification by means of Uniform Manifold Approximation and Projection (UMAP). (C) UMAP plot of malignant cells. (D) UMAP plot depicting the distribution of CytoTRACE scores among malignant cells. (E) The top 10 most positively and negatively stemness‐related genes. (F) Kyoto Encyclopaedia of Genes and Genomes (KEGG) and Gene Ontology (GO) enrichment analyses performed on stemness‐correlated genes. (G) An oncoplot of stemness‐related genes in The Cancer Genome Atlas (TCGA) cohort.

**FIGURE 2:**
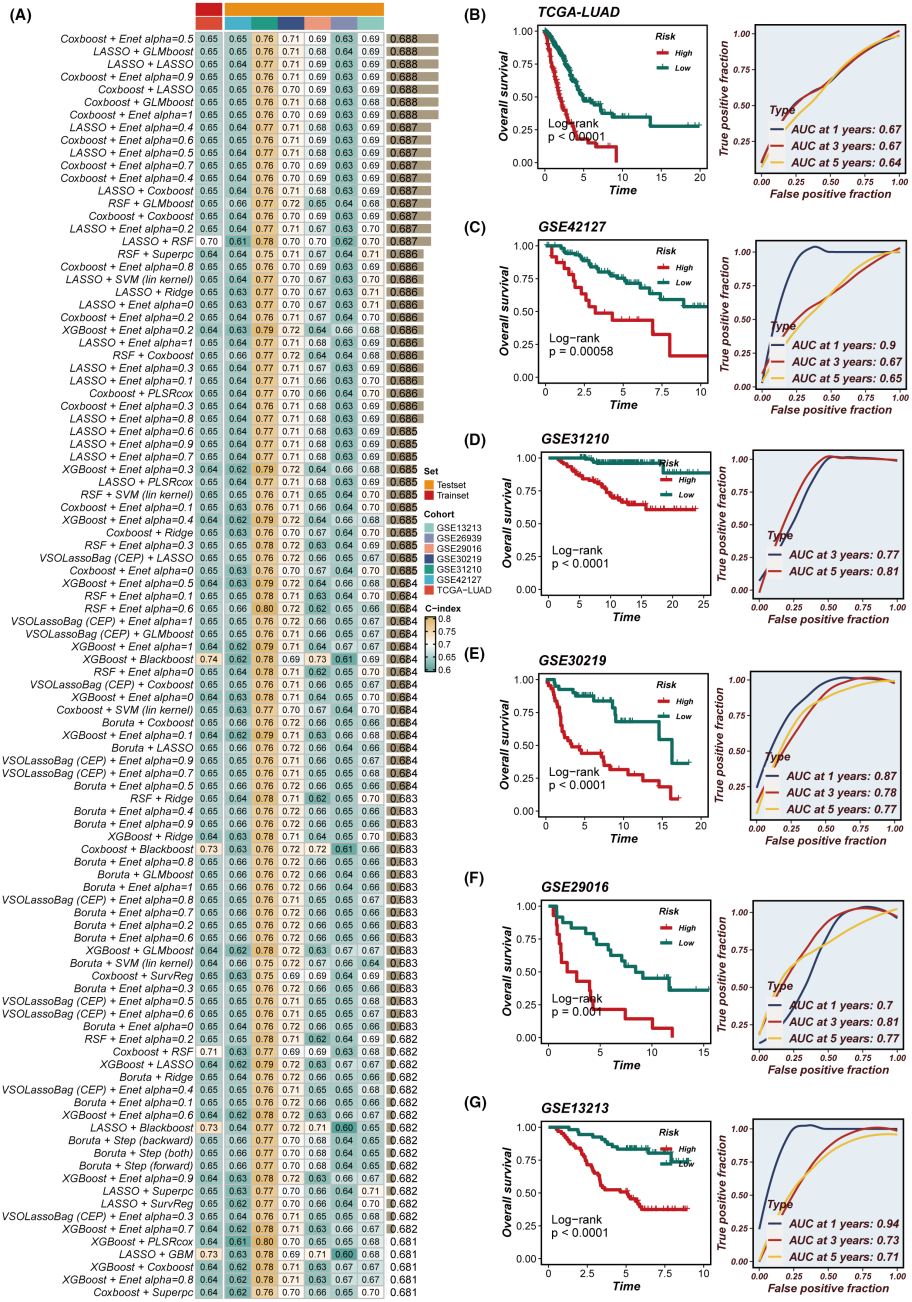
Construction and validation of the signature. (A) Comparison of concordance index (C‐index) values for various algorithm combinations in the seven cohorts. (B‐G) Kaplan–Meier survival analysis for stemness‐related gene signature (SRS) in seven cohorts (left). Area Under Curve (AUC) values of the SRS in predicting 1, 3, and 5‐year survival rates (right).

By performing clustering on scRNA‐seq data, we established a total of 46 clusters (Figure 1A) and proceeded with cell annotation (Figure 1B). Subsequently, we extracted the malignant cells for clustering (Figure 1C) and assigned a stemness score to each group of such malignant cells (Figure 1D). Consequently, we acquired stemness‐related genes (Table S1), and Figure 1E portrays the top 10 most positively and negatively correlated genes. Furthermore, enrichment analyses utilising the Kyoto Encyclopaedia of Genes and Genomes (KEGG) and Gene Ontology (GO) disclosed that these genes participate in a variety of biological processes, including chemical carcinogenesis via reactive oxygen species, endocytosis, and the mitochondrial matrix, as delineated in Figure 2F. The diversity of stemness‐associated gene mutations was likewise assessed in LUAD patients within the TCGA database. Our findings revealed that a substantial proportion, specifically 78.62% (478 out of 608) of LUAD patients, exhibited mutations. Figure 2G displays the top 20 mutations the top 20 mutations in stemness‐related genes are provided, showcasing KRAS as the most frequently mutated gene, accounting for 26% of cases, followed by nineteen others with mutation frequencies ranging between 4% and 16%.

### 3.2 SRS Construction and Validation

Initially, we employed univariate Cox regression analysis within the TCGA cohort to identify pivotal stemness‐related genes with prognostic significance (*p* < 0.01). Furthermore, these genes were required to also satisfy the conditions of differential expression in LUAD tissues (|log2FC| > 0.5 and adjusted *p* < 0.05) within the TCGA cohort. Afterwards, we tested 429 algorithmic combinations on the TCGA data set and calculated the C‐index for each across the validation cohorts. The CoxBoost and Enet integration yielded the highest average C‐index, at 0.688, within the validation cohorts; hence, we selected this combination as our final SRS (Figure 2A). Stratification of LUAD patients into high‐ and low‐risk categories was based on the optimal threshold of SRS score. It was observed that the high‐risk group exhibited a significantly inferior OS compared to the low‐risk group across all cohorts (*p* < 0.05) (Figure 2B–G). Additionally, time‐dependent receiver operating characteristic (ROC) curves affirmed the consistent and reliable prognostic accuracy of SRS within all cohorts (Figure 2B–G).

### 3.3 Comparison of SRS with Other Features

**FIGURE 3:**
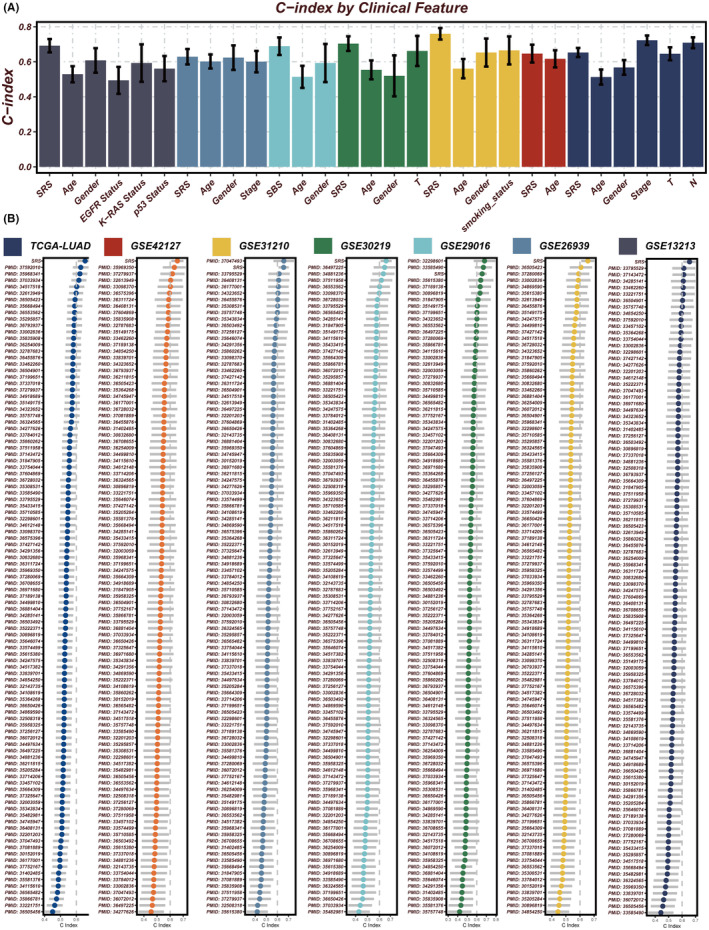
Comparison of SRS with other features. (A) C‐index values of SRS and various clinical features in the seven cohorts. (B) C‐index values of SRS and 101 features in the all cohorts.

Initially, SRS was compared with other clinical features (age, gender, EGFR status, KRAS status, p53 status, stage, T staging, smoking status). The results revealed that the C‐index values of SRS were higher than those of other clinical features, consistently across in the validation cohorts (Figure 3A). Subsequently, SRS was compared with predictive signatures from published studies, and the results demonstrated that the SRS exhibited the best predictive performance across all seven datasets (Figure 3B).

### 3.4 Potential biological peculiarities of the SRS

**FIGURE 4:**
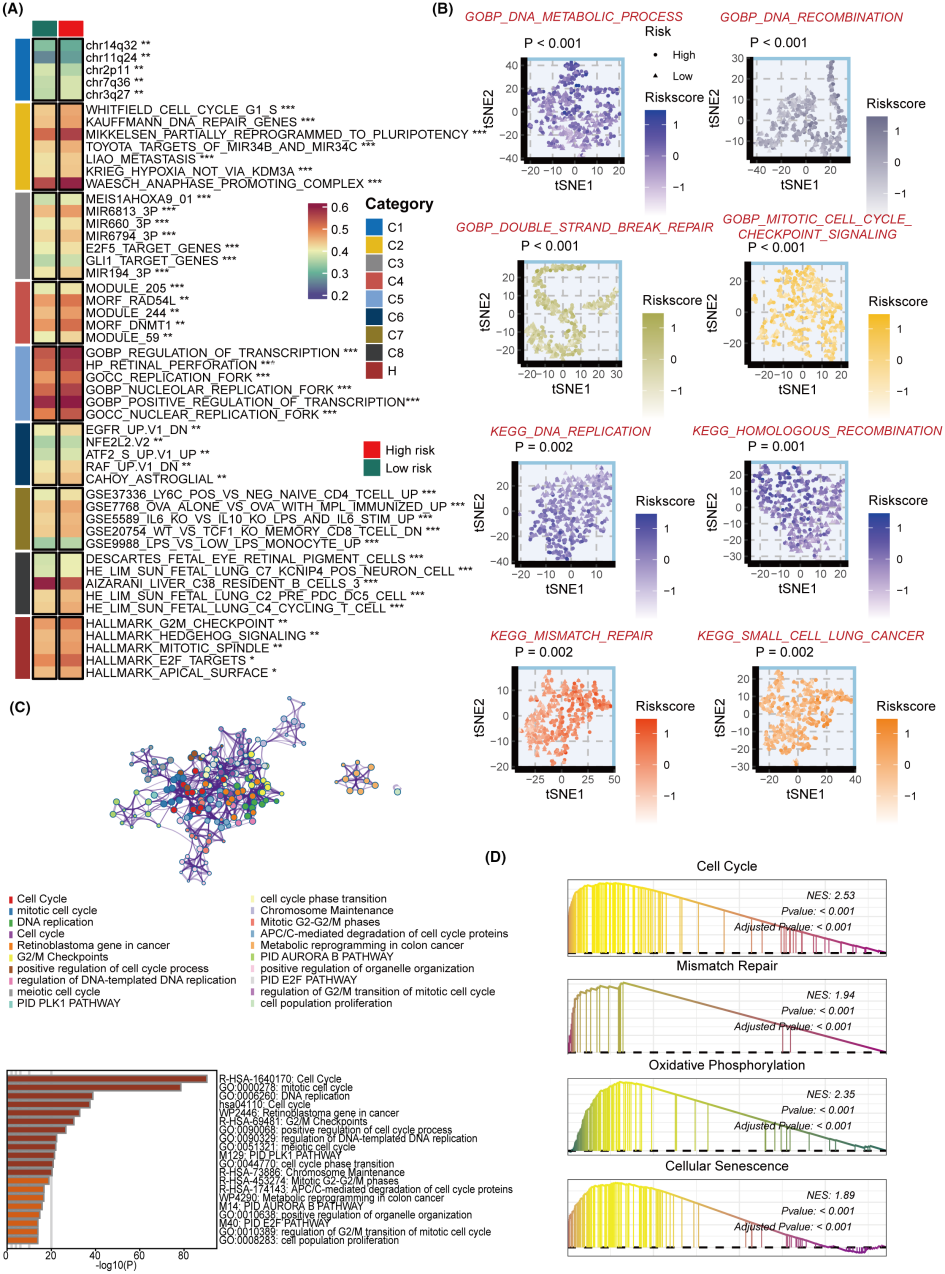
Potential biological peculiarities of the SRS. (A) Gene set variation analysis (GSVA), utilising the Molecular Signatures Database (MsigDB), characterised the biological profiles of two risk groups. (B) t‐distributed stochastic neighbour embedding (t‐SNE) visualisation distinguished the variance in pathway activity associated with GO and KEGG terms between the two risk groups. (C) Metascape‐driven enrichment analysis highlighted the differentially expressed genes between the respective two risk groups. (D) Gene Set Enrichment Analysis (GSEA) of GO and KEGG terms was employed to evaluate the SRS.

To explore the biological mechanisms of SRS, we performed pathway enrichment analysis. We found that the SRS score was strongly correlated with several tumorigenic pathways, such as G2M checkpoint, hedgehog signalling, and E2F targets (Figure 4A). We also observed significant differences in the pathways related to proliferation between the two risk groups (Figure 4B). The differentially expressed genes (DEGs) between the low‐ and high‐risk groups were also enriched in proliferation‐related pathways (Figure 4C). Furthermore, GSEA of KEGG revealed that the high‐risk group was enriched for cell cycle, mismatch repair, oxidative phosphorylation, and cellular senescence. Interestingly, regardless of the enrichment analysis algorithm used, it was found that the SRS score is associated with proliferation‐related pathways. This observation highlights the crucial role of proliferation in maintaining stemness.^38^

### 3.5 Extrinsic immune landscapes of the SRS

**FIGURE 5:**
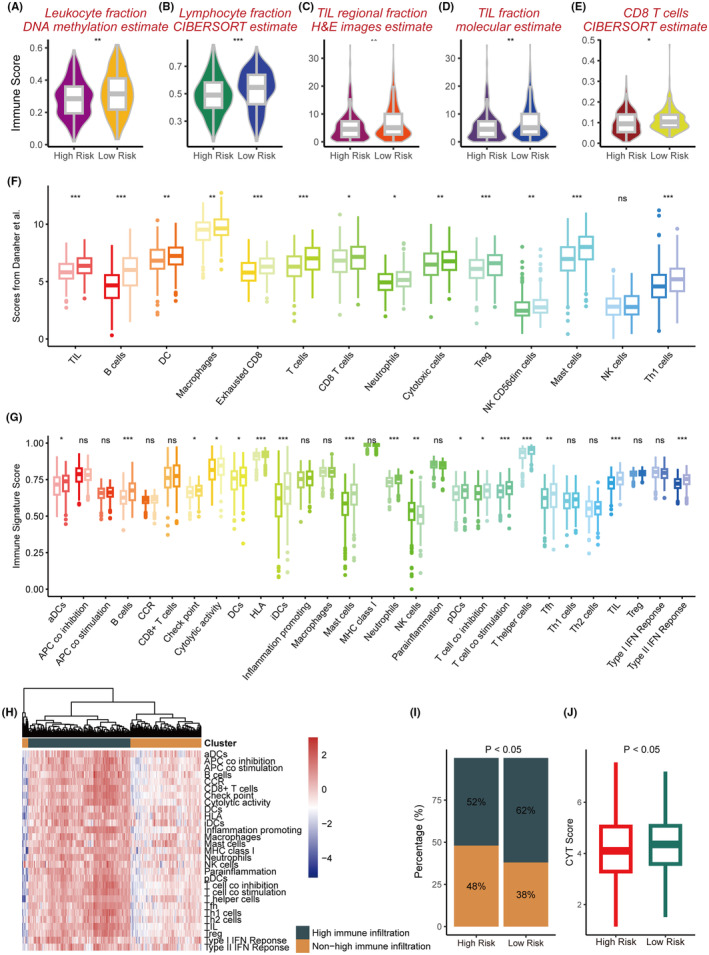
Extrinsic immune landscapes of the SRS. (A) DNA methylation‐based leukocyte abundance analysis comparing the two risk groups. (B) CIBERSORT‐based lymphocyte fraction abundance analysis comparing the two risk groups. (C) Comparison of tumour‐infiltrating lymphocyte (TIL) regional fractions based on estimates from haematoxylin and eosin (H&E) images between the two risk groups. (D) Comparative analysis of TIL abundance using molecular estimates between the two risk groups. (E) Comparison of CD8+ T cells estimated by the CIBERSORT method between the two risk groups. (F) Comparison of 14 immune cells estimated by the Danaher method based on RNA‐sequencing data between the two risk groups. (G) Comparison of the 29 immune signatures estimated by the single sample Gene Set Enrichment Analysis (ssGSEA) method based on RNA‐sequencing data between the two risk groups. (H) Unsupervised clustering based on 29 immune signatures in the cohort from TCGA, yielding two stable immune subtypes. (I) The proportions of high immune infltration and non‐high immune infltration estimated by 29 immune signatures in the high‐risk and low‐risk group. (J) Comparison of cytolytic activity (CYT) score based on estimates from processing diagnostic H&E images between the two risk groups. The significance levels for *p*‐values in (A‐G) are as follows: ^NS^p >0.05, *p < 0.05, **p < 0.01 and ***p < 0.001.

To explore the in‐depth relationship between the immune microenvironment and the SRS, we conducted a multi‐omics investigation of the TCGA cohort. We observed a significant reduction in the proportions of leukocytes, lymphocytes, and particularly tumour‐infiltrating lymphocytes (TILs) within the high‐risk cohort when juxtaposed with the low‐risk cohort (*p* < 0.05) (Figure 5A,B,D). Employing the TIL fraction data per Saltz et al., who utilised deep learning for TIL estimation on H&E‐stained slides, we observed remarkably consistent findings (*p* < 0.05) (Figure 5C). Specifically, the ratio of immune‐stimulatory cells, including CD8+ T cells, was significantly lowered in the high‐risk group relative to the low‐risk group (*p* < 0.05) (Figure 5E). To corroborate these findings via alternative immunological quantification methods, we assessed their distribution in the high‐ and low‐risk groups using the immune infiltration scores of Danaher et al. (Figure 5F) and immune signature scores (Figure 5G). The high‐risk group demonstrated a reduced presence of immune cells such as TILs (*p* < 0.05) (Figure 5F,G). Subsequently, patients in the TCGA cohort were clustered based on immune signature scores through unsupervised clustering. This stratified the cohort into groups with distinct immune infiltration patterns of non‐high and high levels (Figure 5H). Notably, we saw significant overrepresentation of the high‐risk group within the non‐high immune infiltration clusters (*p* < 0.05) (Figure 5I). In addition, tumours with higher risk were associated with significantly diminished CYT scores. Based on these findings, the high‐risk group exhibited a deficiency in immune cells at the tumour site, which may result in poor response to ICI therapy. Additionally, it highlighted that high stemness leads to immune evasion.

### 3.6 Intrinsic immune landscapes of the SRS

**FIGURE 6:**
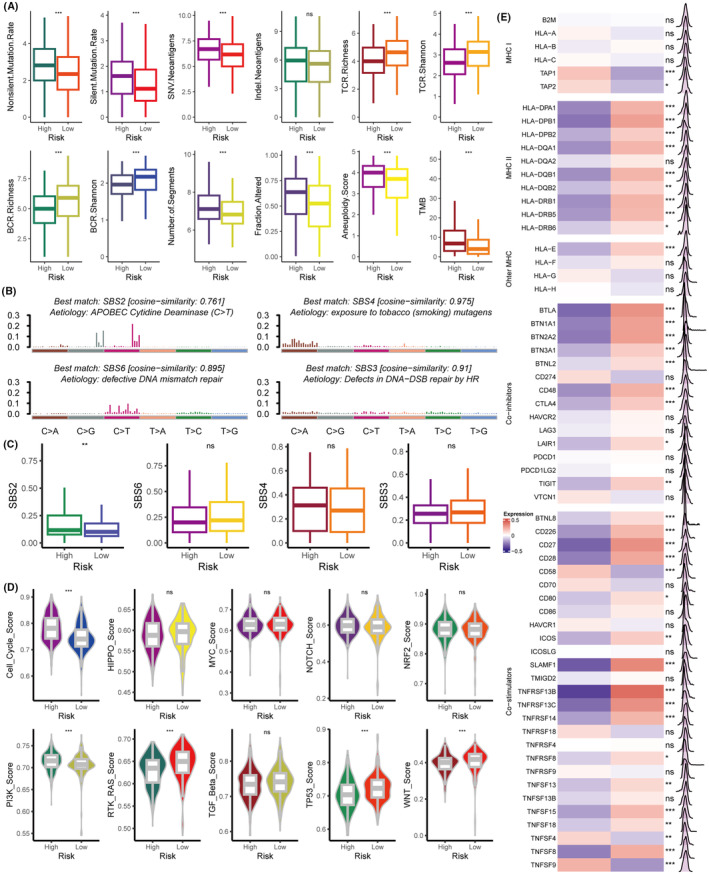
Intrinsic immune landscapes of the SRS. (A) Differential analysis of immunogenomic biomarkers was conducted to delineate disparities between the high‐risk and low‐risk groups. (B) Mutational profiles of four distinct mutational signatures were assessed in terms of their mutational activities. (C) Comparative analysis of these four mutational signatures between the high‐risk and low‐risk groups. (D) Enrichment scores for ten oncogenic pathways were compared to discern differences between the high‐risk and low‐risk groups. (E) Expression comparison of MHC molecules, costimulators, and coinhibitors between high‐risk and low‐risk groups. The significance levels for *p*‐values in (A, C–E) are as follows: ^ns^p >0.05, *p < 0.05, **p < 0.01 and ***p < 0.001.

We initially assessed underlying factors to tumour immunogenicity across the two groups. The high‐risk group was characterised by an elevated mutation rate, neoantigen load, and TMB relative to the low‐risk group (*p* < 0.05) (Figure 6A), as well as significantly reduced TCR and BCR diversity (*p* < 0.05) (Figure 6A). A high TMB enhances immunogenicity, whereas a high SRS score indicates the opposite. To further elucidate the association between immunogenicity and both SRS and TMB, we stratified patients into four subgroups: high SRS/high TMB (HSHT), high SRS/low TMB (HSLT), low SRS/high TMB (LSHT), and low SRS/low TMB (LSLT), with a TMB cutoff set at 10. Upon comparing immune cell abundances, the LSHT group emerged with the highest levels of lymphocytes, whereas the HSLT group exhibited the lowest (*p* < 0.05) (Figure S1). A high SRS score was found to impede immunogenicity, and a low TMB level correlated with reduced immunogenicity. As surmised, diminished infiltration of lymphocytes was observed in both HS and LT (*p* < 0.05) (Figure S1). It stands to reason that their co‐presence (HSLT) culminates in a tumour microenvironment (TME) with the sparsest infiltration of these immune cells. Conversely, the LSHT might foster the most abundant presence of lymphocytes within the TME. However, the immunogenicity of the remaining two subgroups (HSHT and LSLT) appears more contentious due to each harbouring an immune‐suppressing (HS or LT) and an immune‐enhancing (LS or HT) element. Subsequent analyses revealed that the LSLT subgroup contained higher levels of lymphocytes compared to HSHT (*p* < 0.05) (Figure S1). In summary, the hierarchy of immunogenicity from most to least pronounced is: LSHT > LSLT > HSHT > HSLT. Therefore, tumours with low SRS score presented with signifcantly better immunogenicity than those with high SRS score regardless of TMB level. To gain insight into the mutational processes within the high‐ and low‐risk groups, we identified mutational signatures from somatic mutation profiles, thereby delineating four distinct mutagenesis patterns in the TCGA cohort (Figure 6B). Notably, the high‐risk group exhibited a significant upsurge in the SBS2 (APOBEC cytidine deaminase) signature compared to the low‐risk group (*p* < 0.05) (Figure 6C). APOBEC mutagenesis has been shown contributes to cancer progression by promoting subclonal heterogeneity, immune evasion, and genomic instability, thereby enhancing tumour survival and resistance to therapy.^39^ Additionally, we computed enrichment scores for oncogenes across ten canonical oncogenic pathways for both groups. The high‐risk group demonstrated elevated scores in the cell cycle and PI3K pathways, whereas the RAS, Wnt, and TP53 pathways were significantly more prevalent in the low‐risk group (*p* < 0.05) (Figure 6D). These results reinforce earlier evidence that implicates the PI3K and cell cycle pathways as critical for CSC survival and propagation.^38^, ^40^

The high‐risk group was also found to express decreased levels of MHC class II‐related antigen‐presenting molecules compared to the low‐risk group (most *p* < 0.05), suggesting an intrinsic immune evasion mechanism (Figure 6E). Furthermore, we observed that immune checkpoint molecules (such as CTLA4) and costimulatory molecules were more abundantly expressed in the low‐risk group (most *p* < 0.05) (Figure 6E). From these observations, we deduced that these immune checkpoint molecules are involved in eliciting a response to ICI therapy.

### 3.7 Validation of the SRS as a predictive biomarker for immunotherapy response

**FIGURE 7:**
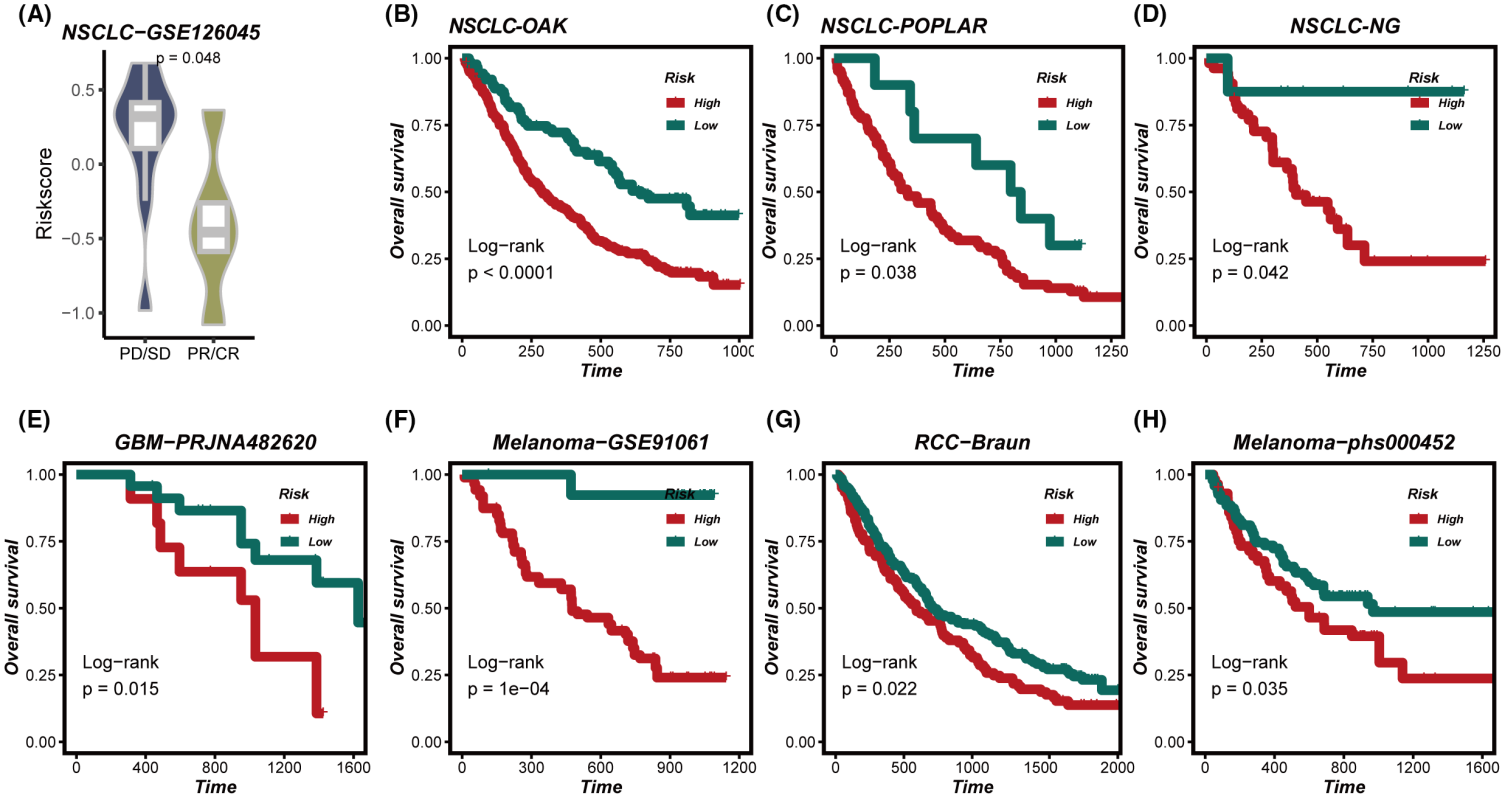
Validation of the SRS as a predictive biomarker for immunotherapy response. (A) Association of SRS and immunotherapy responses in GSE126045 dataset. (B‐H) Kaplan–Meier survival analysis of SRS for overall survival (OS) in the OAK, POPLAR, NG, PRJNA482620, GSE91061, Braun and phs000452 cohorts.

Building on the prognostic capability of the SRS for immunotherapy efficacy, we proceeded to validate its utility across several datasets. In the GSE126045 dataset, patients with low SRS score demonstrated improved responses to immunotherapy (*p* < 0.05) (Figure 7A). Moreover, patients with low SRS score in datasets OAK, POPLAR, NG, PRJNA482620, GSE91061, Braun, and phs000452 exhibited enhanced survival rates (all *p* < 0.05) (Figure 7B–H).

### 3.8 Experimental validation of CKS1B

**FIGURE 8:**
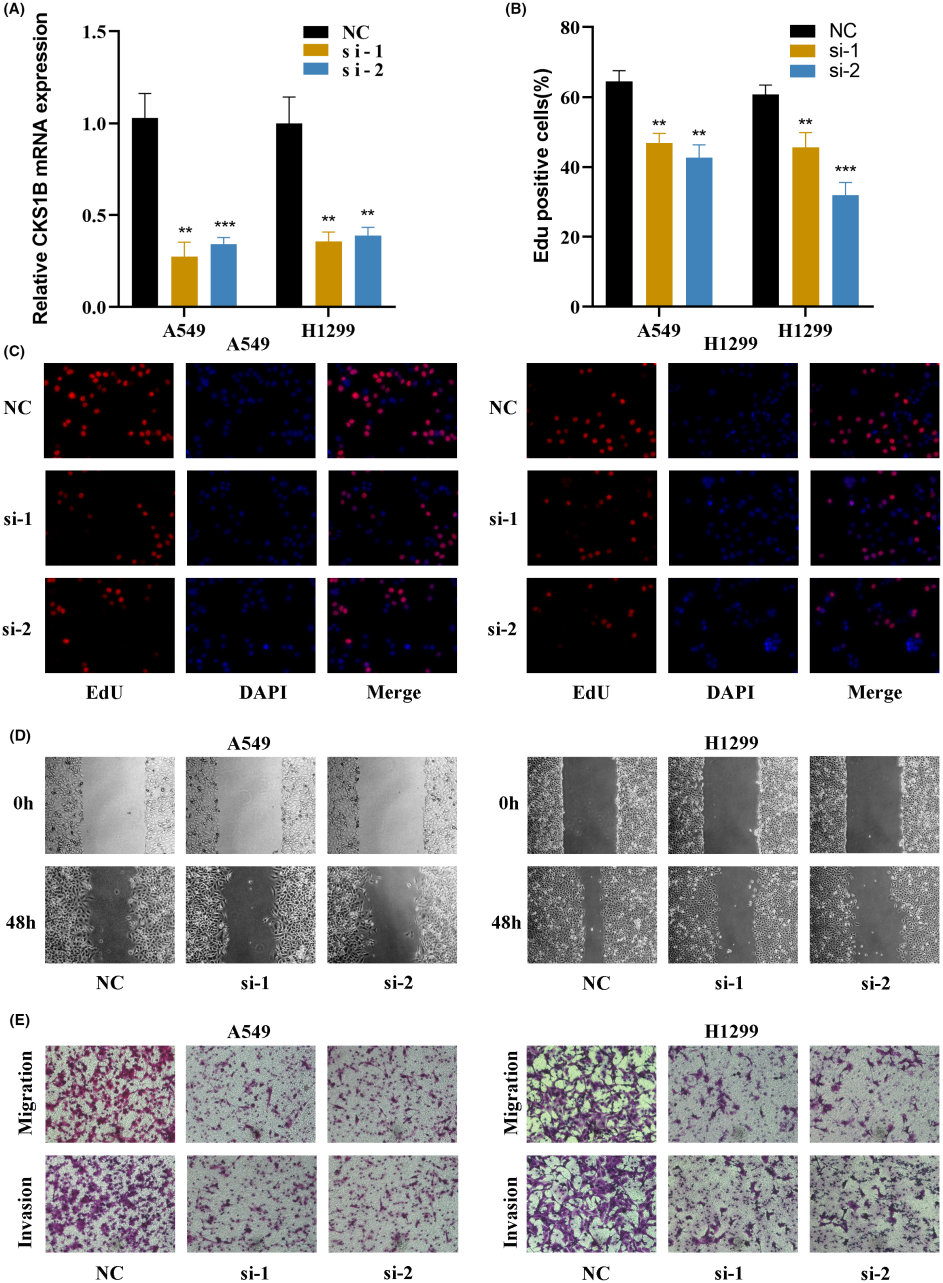
Role of CKS1B in Regulating Proliferation of LUAD Cells. (A) Efficiency of CKS1B knockdown in A549 and H1299 LUAD cell lines. Specific siRNAs were employed to selectively reduce CKS1B expression. (B, C) Impact of CKS1B knockdown on cell proliferation in LUAD cells. The 5‐ethynyl‐2′‐deoxyuridine (EdU) incorporation assay was utilised to evaluate cellular proliferation rates following CKS1B suppression. Quantitative analysis reveals a significant decrease in EdU‐positive cells in CKS1B‐depleted A549 and H1299 cells, indicating a reduction in DNA synthesis and cell proliferation. (D) Effects of CKS1B knockdown on cell migration assessed by wound healing assays. Images captured at 0 and 24 hours post‐wounding illustrate delayed wound closure in CKS1B‐silenced cells compared to control siRNA‐treated cells, highlighting the role of CKS1B in facilitating cell migration. (E) Reduction in cell invasion and migration capabilities upon CKS1B knockdown, as determined by Transwell assays. The decrease in the number of cells traversing the membrane in both invasion (coated with Matrigel) and migration assays substantiates the critical involvement of CKS1B in promoting LUAD cell invasiveness and motility. The significance levels for *p*‐values in (A, B) are as follows: **p < 0.01 and ***p < 0.001.

**FIGURE 9:**
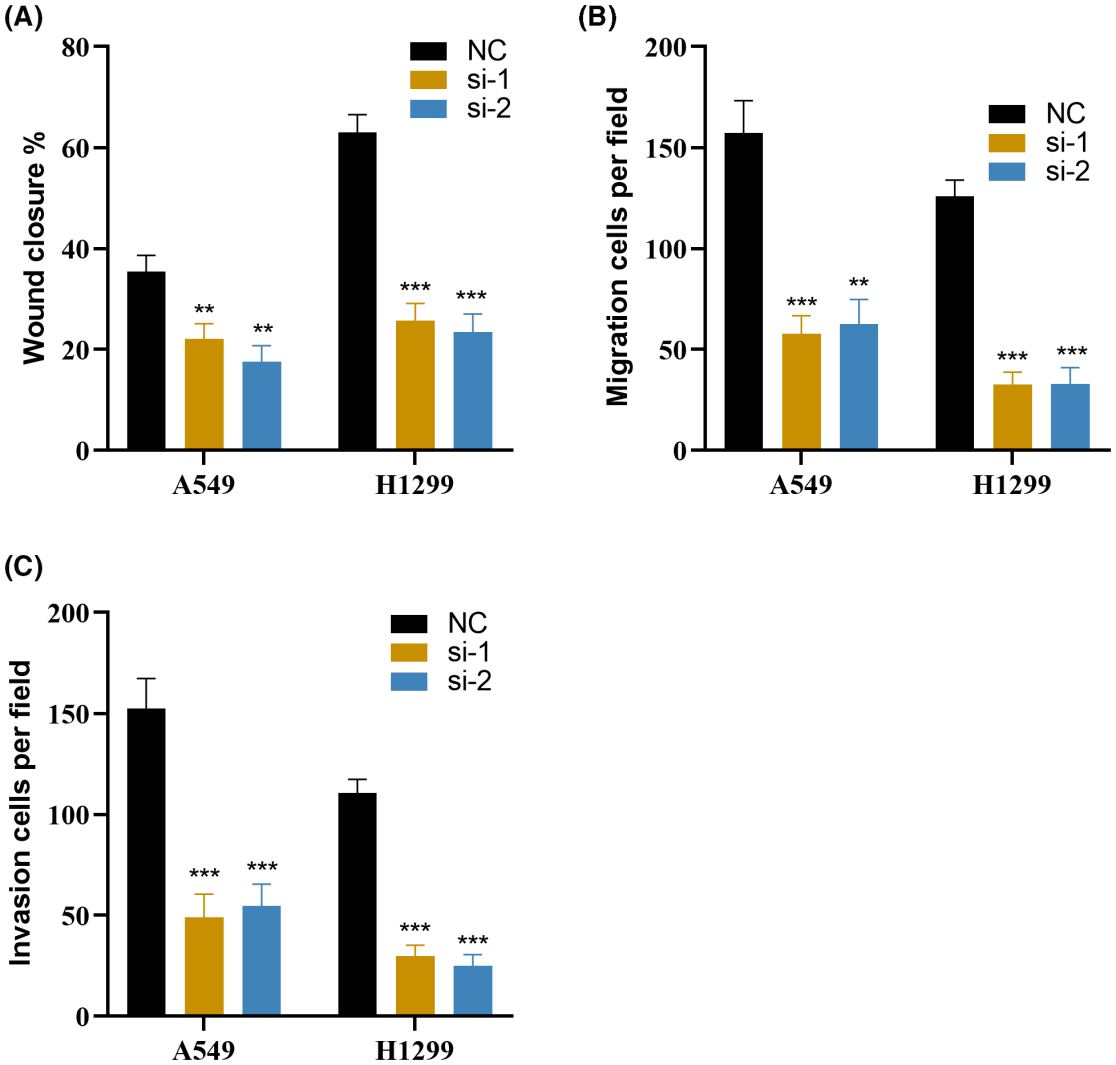
Experimental validation of CKS1B (A–C) Quantitative analysis demonstrates that knockdown of CKS1B leads to a deceleration in wound healing, accompanied by diminished invasive and migratory capacities in lung adenocarcinoma cells. The significance levels for *p*‐values in (A–C) are as follows: **p < 0.01 and ***p < 0.001.

Based on the Enet algorithm, CKS1B was identified as the most significant gene in SRS. To investigate the pivotal role of CKS1B in the pathogenesis of LUAD, we modulated CKS1B expression in A549 and H1299 cell lines through targeted siRNA‐mediated knockdown (Figure 8A). Subsequently, EdU incorporation assays were employed to assess the impact of CKS1B knockdown on cellular proliferation rates. Our findings from these assays demonstrate a considerable diminution in proliferative capabilities of LUAD cells post‐CKS1B knockdown (Figure 8B,C). Further exploratory analyses were conducted to delineate the effects of CKS1B suppression on the migratory and invasive properties of LUAD cells. Wound healing assays provided quantitative evidence that CKS1B knockdown significantly retards the process of wound closure, indicative of a marked reduction in cell migration (Figures 8D and 9A). Moreover, using Transwell migration and invasion assays, we observed a pronounced decrease in the invasive and migratory potential of LUAD cells following CKS1B knockdown, reinforcing the hypothesis of CKS1B's crucial involvement in the metastatic phenotype of LUAD cells (Figures 8E and 9B,C). Collectively, these results elucidate a critical function of CKS1B in promoting LUAD cell proliferation, migration, and invasion, thereby highlighting its potential as a therapeutic target in LUAD treatment strategies.

## 4 DISCUSSION

Immunotherapy stands as a transformative approach to cancer treatment, functioning by activating or augmenting the patient's immune response to recognise and eradicate cancer cells, while minimising collateral damage to healthy cells simultaneously.^41^ Immunotherapy, widely implemented in managing diverse malignancies such as kidney cancer, endometrial cancer, lung cancer, and nasopharyngeal carcinoma, has attained substantial efficacy, often accompanied by a reduced incidence of adverse effects.^42^, ^43^, ^44^, ^45^ Notably, clinicians have observed that only a subset of patients exhibit responsiveness to immunotherapy. Presently, researchers suggest that this variability in response may be attributable to factors such as tumour type, the patient's immune status, and additional variables, yet the biological underpinnings of this phenomenon continue to elude full understanding.^46^, ^47^ Single‐cell sequencing technology boasts distinct strengths in unveiling tumour cell heterogeneity, pinpointing therapeutic targets, and tailoring personalised treatment strategies, as corroborated by an array of prior investigations.^48^, ^49^

In this study, by analysing 208,506 LUAD single cells, we identified 552 stemness‐related genes. Subsequently, based on nine key stemness‐related genes with prognostic value (CKS1B, GAPDH, PPIA, KRT8, TXN, S100A16, YWHAZ, PERP, SEC61G), we employed the Enet to construct the SRS with superior predictive potency for LUAD prognosis, and validated this signature in seven independent cohorts, all demonstrating excellent predictive accuracy. Additionally, we leveraged the comprehensive TCGA dataset to investigate cancer responsiveness to immunotherapy. We discerned that the low‐risk group manifested an inflammatory immune response pattern, exemplified by elevated CD8+ T cell infiltration ascertained through the CIBERSORT methodology. Utilising the immune infiltration scores developed by Danaher et al., along with the ssGSEA for the determination of immune cell infiltration levels in cancers, we observed that the immune score was notably higher in the low‐risk group compared to the high‐risk group. This further authenticated the intensified anti‐tumour immune activity prevalent in the low‐risk cohort. The density of TILs has been positively linked to immune responsiveness in patients with diverse cancer types in multiple investigations.^50^ Therefore, it seems that the activation of anti‐tumour immunity, diminished cancer stemness, and heightened tumour immunogenicity might account for the increased likelihood of the low‐risk group deriving greater benefit from immunotherapy. This highlights the potential for using our SRS not only as a prognostic tool but also as a predictive marker for immunotherapy efficacy.

Based on the Enet algorithm, CKS1B was identified as the most significant gene in SRS. Studies have shown that high expression of CKS1B may be associated with abnormal proliferation of tumour cells, malignant transformation, invasiveness, and prognosis.^51^, ^52^, ^53^ Li and colleagues found that CKS1B expression in pancreatic tumour tissues was higher than in normal tissues, and this result was validated by reverse transcription‐quantitative polymerase chain reaction. Moreover, immune infiltration analysis revealed that CKS1B expression was significantly associated with the level of immune cell infiltration in pancreatic cancer, and knockdown experiments showed a strong correlation between CKS1B and the cell activity and invasiveness of pancreatic cancer.^54^ A predictive model developed by Mohamad and others, based on five genes including CKS1B, CCT2, PRKDC, NONO, and UBE2A, successfully predicted the prognosis of patients with multiple myeloma and has been validated in several independent datasets. These results suggest that CKS1B could play a crucial role in the development of malignant tumour. However, there is still a gap in the study of CKS1B in LUAD. Based on a series of knockout experiments, it is more confirmed that aberrant expression of CKS1B in LUAD can contribute to cell proliferation, migration, and invasion.

Although this study advances the clinical comprehension of the SRS, it is not devoid of limitations. The cohorts display heterogeneity as a result of employing varied sequencing and microarray technologies, a variance we endeavoured to reconcile through standard normal transformations, though with limited success. Additionally, the retrospective nature of our samples highlights the imperative for validation within a future, larger prospective cohort. Lastly, the role of CKS1B in the regulation of cancer cell stemness remains to be conclusively determined and warrants additional experimental corroboration through in vivo and in vitro investigations.

In conclusion, our study illuminates the critical role of stemness‐related genes in predicting LUAD prognosis and offers significant insights into the immunological landscape of these tumours. The SRS we developed holds promise for guiding personalised cancer treatment strategies, potentially leading to more effective and tailored therapeutic interventions for patients with LUAD.

## AUTHOR CONTRIBUTIONS

**Bicheng Ye:** Data curation (equal); methodology (equal); writing – original draft (equal); writing – review and editing (equal). **Ge Hongting:** Data curation (equal); methodology (equal); writing – original draft (equal); writing – review and editing (equal). **Wen Zhuang:** Data curation (equal); methodology (equal); writing – original draft (equal); writing – review and editing (equal). **Cheng Chen:** Data curation (supporting); funding acquisition (supporting); methodology (equal). **Shulin Yi:** Data curation (supporting); methodology (supporting). **Xinyan Tang:** Investigation (supporting); validation (supporting). **Aimin Jiang:** Formal analysis (supporting); investigation (supporting). **Yating Zhong:** Conceptualization (lead); writing – review and editing (lead).

## FUNDING INFORMATION

This work was supported by the key university fund of Yangzhou Polytechnic College.

## CONFLICT OF INTEREST STATEMENT

The authors declare no potential conflicts of interest.

## Supporting information


Figure S1.

Table S1.

## Data Availability

The datasets supporting the findings of this study are available in the TCGA (https://gdc.xenahubs.net) and GEO (https://www.ncbi.nlm.nih.gov/geo/) databases. Further inquiries can be directed to the corresponding author.
