# Supplementary figures and images for: Deciphering lung adenocarcinoma prognosis and immunotherapy response through an AI‐driven stemness‐related gene signature
Source: J Cell Mol Med. 2024 Jul 24;28(14):e18564. doi: 10.1111/jcmm.18564 (PMC11268368; doi:10.1111/jcmm.18564)

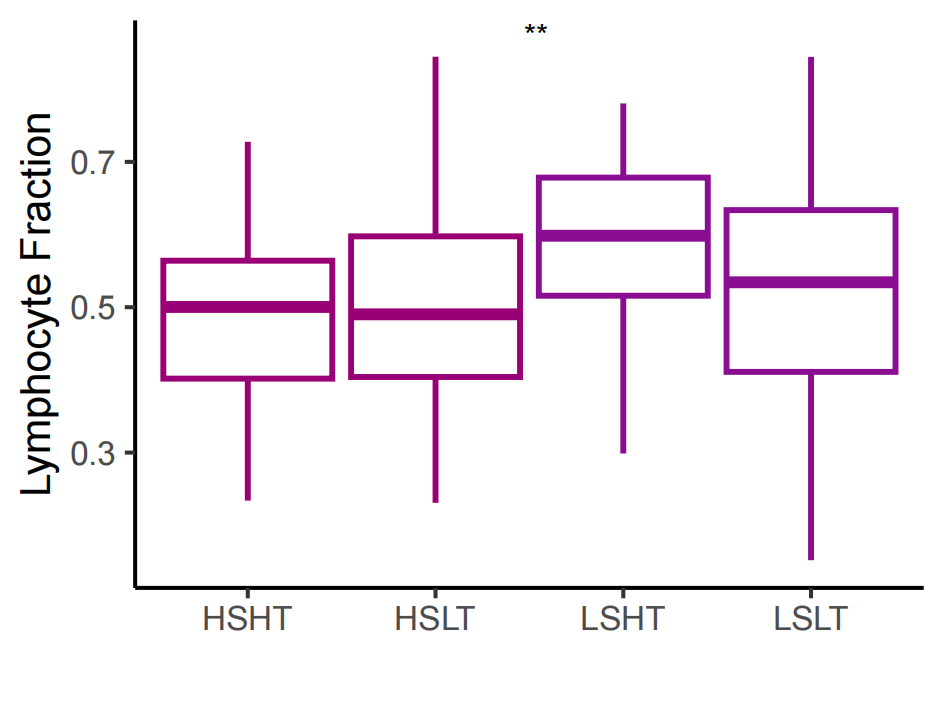

Supplement: Supplementary file 1 — Figure S1. [file JCMM-28-e18564-s002.tif]
